# A minority of somatically mutated genes in pre‐existing fatty liver disease have prognostic importance in the development of NAFLD

**DOI:** 10.1111/liv.15283

**Published:** 2022-05-11

**Authors:** Jake P. Mann, Matthew Hoare

**Affiliations:** ^1^ Institute of Metabolic Science University of Cambridge Cambridge UK; ^2^ School of Clinical Medicine University of Cambridge Cambridge UK; ^3^ CRUK Cambridge Institute University of Cambridge Cambridge UK

**Keywords:** alcohol‐related liver disease, genomic analysis, GPAM, NAFLD, precision medicine, variation

## Abstract

**Background:**

Understanding the genetics of liver disease has the potential to facilitate clinical risk stratification. We recently identified acquired somatic mutations in six genes and one lncRNA in pre‐existing fatty liver disease. We hypothesised that germline variation in these genes might be associated with the risk of developing steatosis and contribute to the prediction of disease severity.

**Methods:**

Genome‐wide association study (GWAS) summary statistics were extracted from seven studies (>1.7 million participants) for variants near *ACVR2A*, *ALB*, *CIDEB*, *FOXO1*, *GPAM*, *NEAT1* and *TNRC6B* for: aminotransferases, liver fat, HbA1c, diagnosis of NAFLD, ARLD and cirrhosis. Findings were replicated using GWAS data from multiple independent cohorts. A phenome‐wide association study was performed to examine for related metabolic traits, using both common and rare variants, including gene‐burden testing.

**Results:**

There was no evidence of association between rare germline variants or SNPs near five genes (*ACVR2A*, *ALB*, *CIDEB*, *FOXO1* and *TNRC6B*) and risk or severity of liver disease. Variants in *GPAM* (proxies for p.Ile43Val) were associated with liver fat (*p* = 3.6 × 10^−13^), ALT (*p* = 2.8 × 10^−39^) and serum lipid concentrations. Variants in *NEAT1* demonstrated borderline significant associations with ALT (*p* = 1.9 × 10^−11^) and HbA1c, but not with liver fat, as well as influencing waist‐to‐hip ratio, adjusted for BMI.

**Conclusions:**

Despite the acquisition of somatic mutations at these loci during progressive fatty liver disease, we did not find associations between germline variation and markers of liver disease, except in *GPAM.* In the future, larger sample sizes may identify associations. Currently, germline polygenic risk scores will not capture data from genes affected by somatic mutations.

Lay Summary

We have recently found recurrent genetic mutations in the liver of patients with end‐stage fatty liver disease. We believe these mutations develop to protect liver cells from damage. However, when we study very large numbers of patients, hereditary genetic mutations at the same places in DNA, do not seem to affect the risk that a person will develop fatty liver disease in the first place.

## 1 INTRODUCTION

Non‐alcoholic fatty liver disease (NAFLD) affects around 25% of the worldwide population and is emerging as the fastest‐growing form of liver disease in developed countries.^1^, ^2^ It encompasses a spectrum of diseases from simple hepatic steatosis, through non‐alcoholic steatohepatitis (NASH) to cirrhosis, with the attendant risks of liver failure and hepatocellular carcinoma (HCC). Hepatic steatosis is strongly associated with insulin resistance, obesity and other features of the metabolic syndrome, which has led to the development of the term metabolic dysfunction‐associated fatty liver disease (MAFLD).^3^ Given the number of patients at risk of development of NAFLD and subsequent health problems, there is an urgent need to stratify patients based upon long‐term risks, so that even countries with well‐resourced healthcare schemes can cope with the patient numbers predicted to develop end‐stage liver disease over the next few decades.

At present, there are a number of clinically deployed non‐invasive tests, including elastography and serum biomarkers that can predict levels of hepatic fibrosis and long‐term risk in NAFLD. Significant interest has been generated in the potential of germline genetic variation in the pathogenesis and prognostication of NAFLD.^4^ Single‐nucleotide polymorphisms (SNPs) near several genes, including *PNPLA3*,^5^ *HSD17B13*,^6^ *TM6SF2* ^7^ and *MBOAT7*,^8^, ^9^ have been shown to be associated with long‐term risk of several disease‐related outcomes in NAFLD,^10^ as well as in alcohol‐related liver disease (ARLD),^11^ through genome‐wide association studies (GWAS). Their mechanistic contribution to fatty liver disease initiation or progression is only starting to become clear. These variants are common but associated with small effect sizes, when studied in isolation. This has suggested the potential for combining multiple genotypes into a polygenic risk score: the analysis of several SNPs in patients at baseline as a predictive tool for long‐term outcomes in both NAFLD^12^ and ARLD.^11^

Similarly, other studies have focused on rare pathogenic germline variants, such as in *APOB* (encoding apolipoprotein B), that lead to NAFLD with high penetrance.^13^ Although these cases can inform us about underlying disease pathogenesis, their low frequency suggests that they are unlikely to be informative in the stratification of most patients within the general NAFLD population.

We have recently identified recurrent somatic mutations within the liver of patients with NAFLD and ARLD^14^ Recurrent non‐synonymous mutations were seen in six coding genes: *ACVR2A*, *ALB*, *CIDEB*, *FOXO1*, *GPAM*, *TNRC6B*, plus the long non‐coding (lnc)RNA *NEAT1*. These mutations were found in multiple hepatocyte clones throughout the liver, with evidence of convergent evolution: the identification of independent clones with mutations in the same genes, suggesting strong selection pressure to acquire these variants during progressive fatty liver disease. Many of these variants were predicted to result in an absence of functional protein. Amongst these genes, three are involved in lipid metabolism suggesting a potential functional role in disease pathogenesis: *CIDEB*, mediates fusion and cargo transfer of cytoplasmic lipid droplets^15^; *FOXO1*, the main transcription factor downstream of insulin,^16^ but also a major regulator of lipid metabolism^17^ and; *GPAM*, encoding GPAT1, the enzyme catalysing the initial step in triglyceride synthesis.^18^ Through functional validation of the hot‐spot mutations in *FOXO1*, we identified that this impacted the response to insulin, glycolysis and lipid metabolism. Although promising for improving the mechanistic understanding of disease pathogenesis, our current evidence only implicates them as acquired mutations developing in pre‐existing fatty liver disease. However, the prognostic or therapeutic role that these acquired mutations could play in NAFLD and ARLD remains unknown.

As there was strong selective pressure to acquire these mutations in the context of pre‐existing NAFLD, we were interested to explore whether common SNPs or rare germline variation near these recurrently mutated genes might be associated with the development of fatty liver and liver‐related outcomes in NAFLD. Data from well‐established variants have shown that hepatic fat accumulation has been causally linked to clinical liver events (e.g. hepatocellular carcinoma).^12^ We hypothesised that germline variation providing weaker modulation of disease phenotypes would exist and might improve the performance of polygenic risk scores being developed for prognostication in NAFLD and ARLD.

Amongst these genes, germline coding variants at *GPAM* have been associated with serum ALT levels in an exome‐wide association study of the UK Biobank cohort,^19^ particularly p.Ile43Val. Further study found that these variants were also associated with hepatic fat content and histological markers of liver damage in independent NAFLD cohorts. Other studies using GWAS have replicated these findings in further cohorts,^20^, ^21^, ^22^ where rs10787429 in *GPAM* was associated with elevated ALT in both NAFLD and ARLD. Here we use data from multiple GWAS to investigate whether similar associations are observed for the other five recurrently mutated genes and one recurrently mutated locus.

## 2 METHODS

### 2.1 Identification of genes enriched for somatic mutations in ARLD and NAFLD

As described in detail elsewhere,^14^ whole‐genome sequencing was performed on diseased, non‐malignant hepatic tissue from patients with NAFLD and ARLD undergoing tumour resection or liver transplant. The dN/dScv method^23^ was used to identify six coding genes with higher numbers of nonsynonymous mutations relative than expected.

### 2.2 Annotation of somatic mutants and genomic regions

After removal of duplicates, somatic mutations at *ACVR2A*, *ALB*, *CIDEB*, *FOXO1*, *GPAM* and *TNRC6B*, plus one non‐coding lncRNA *NEAT1* ^14^ were annotated with predicted consequence, impact on transcript and prevalence within the 1000 Genomes dataset^24^ (*n* = 2504), Exome Sequencing Project^25^ (*n* = 6503) and gnomAD^26^ (*n* = 141 456) using the Ensembl Variant Effect Predictor.^27^ All non‐synonymous single nucleotide variants were also annotated with predicted functional consequences using dbNSFP.^28^

For the variants that had previously been identified in any of the above population genomic sequencing datasets, we searched Phenoscanner^29^ and the Common Metabolic Disease Portal^30^ for any evidence of association with metabolic traits. No data were available on Phenoscanner for these ultra‐rare variants. The Common Metabolic Disease Portal search yielded 96 variant‐trait associations: therefore, rather than apply genome‐wide association significance cut‐off, the critical *p*‐value for significance for this analysis only was *p* < 5.2 × 10^−4^ (i.e. 0.05/96).

In addition, we annotated each of the six genes with their expected and observed number of predicted loss of function (pLoF) and missense mutations from gnomAD.^26^ In brief, the ratio between observed and expected pLoF mutants is an indicator of each gene's tolerance to haploinsufficiency. Such that a gene with a low observed/expected ratio (defined as the upper 95% confidence interval <0.35) has fewer pLoF mutants than other genes, suggesting that humans are relatively intolerant of haploinsufficiency.

### 2.3 Association with markers of liver disease from genome‐wide association studies

Summary statistics from genome‐wide association studies (GWAS) were searched for all variants within the genomic regions for the seven regions of interest (Table S1). Regions included were (GRCh37): *ACVR2A*: chr2:148602086‐148 688 393; *ALB*: chr4:74262831‐74 287 129; *CIDEB*: chr14:24774302‐24 780 636; *FOXO1*: chr13:41129804‐41 240 734; *GPAM*: chr10:113909624‐113 975 135; *NEAT1*: chr11:65190245‐65 213 011; *TNRC6B*: chr22:40440821‐40 731 812.

In addition, for comparison, we extracted summary statistics for four well‐validated genome‐wide significance risk variants for fatty liver disease: rs738409C > G in *PNPLA3*,^5^ rs58542926C > T in *TM6SF2*,^7^ rs2642438A > G in *MTARC1* ^31^ and rs72613567TA > T in *HSD17B13*.^6^ However, for some studies data on rs72613567TA > T were not available therefore we used rs13125522A > G as a proxy, which is in strong linkage disequilibrium (*R* ^2^ = 0.97) with rs72613567.^32^

Summary statistics were obtained from the Pan‐UK BioBank analysis^33^ for alanine aminotransferase (ALT), aspartate aminotransferase (AST), glycosylated haemoglobin (HbA1c), diagnosis of NAFLD (phecode‐571.5), diagnosis of ARLD (phecode‐317.11), liver fibrosis (icd10‐K74), cirrhosis (phecode‐571) and other liver diseases (phecode‐571.5).

We also obtained summary statistics for diagnosis of NAFLD from Anstee et al.^34^ and MRI liver fat from Liu et al.,^35^ which utilises UK BioBank data. These data were accessed through GWAS Catalogue.^36^ For replication of findings, we first obtained categorical data on liver‐related diagnoses from the FinnGen study: NAFLD, ARLD, hepatocellular carcinoma and intrahepatic cholangiocarcinoma and cirrhosis. For replication of observations for ALT, we obtained data from BioBank Japan^37^ and Pazoki et al.^38^ For replication of findings for HbA1c, we obtained summary statistics on HbA1c from the MAGIC consortium (trans‐ancestry meta‐analysis by Chen et al.^39^) and BioBank Japan, plus diagnosis of type 2 diabetes mellitus (T2DM) in East Asian individuals from Spracklen et al.^40^ The total number of unique participants included from these GWAS summary statistics was 1 628 945.

All variants from the above regions were extracted, and coordinates from FinnGen were carried over from GRCh38 to GRCh37 using the Ensembl Assembly Converter. Manhattan plots were produced for each trait, illustrating only variants within the regions of interest. Significance was defined as *p* < 5 × 10^−8^. Within regions that had variants with a significant association, we used FIVEx to look for expression quantitative trait loci (eQTL), which extracts data from the European Bioinformatic Institute eQTL Catalogue.^41^

### 2.4 Gene‐based phenome‐wide association study for common variants

We sought to explore associations between germline variation in the coding genes and lncRNA of interest and metabolic traits using a phenome‐wide association study approach. Phenoscanner^29^ and the Common Metabolic Diseases Knowledge Portal^30^ were searched for each of the six coding genes plus *NEAT1*. Rare variants (mean allele frequency [MAF] <0.01) were excluded and results were filtered for traits relevant to ARLD and NAFLD. Significance was defined as *p* < 5 × 10^−8^. Data on eQTLs were obtained using the Qtlizer package for R^42^ for all significant associations.

We also searched for any metabolite‐wide associations within the regions of interest using data from Lotta et al.^43^ (https://omicscience.org/apps/crossplatform/); however, we did not identify any significant (*p* < 4.9 × 10^−10^, as defined by the authors) associations.

### 2.5 Association between rare coding variants and traits related to NAFLD or ARLD

We next investigated whether rare coding variants individually, or in combination using gene‐burden analyses, were associated with markers of liver disease or related metabolic traits. We used data from https://genebass.org/ ^44^ and https://azphewas.com/,^45^ which derive data from the UK BioBank 300 k Exomes. These analyses were not available for the lncRNA *NEAT1*. Extracted associations for individual variants, and gene‐burden tests for predicted loss of function (pLoF), missense and synonymous variants. Significance, as defined by the original studies, was *p* < 2.5 × 10^−8^ for GeneBass (using SKAT‐O test) and *p* < 2.0 × 10^−9^ (−log10[8.7]) for AZPheWAS.

### 2.6 Analyses

Linkage disequilibrium between variants, including the previously reported rs2792751T > V (p.Ile43Val) in GPAM, was calculated using SNiPA.^46^

Data were analysed using R 4.0.2^47^ and the code used in the analyses is available from https://doi.org/10.5281/zenodo.4656979.

## 3 RESULTS

We have recently identified recurrent somatic mutations in non‐malignant liver tissue from individuals with ARLD and NAFLD through laser capture microdissection and whole‐genome sequencing.^14^ Through this approach we identified six coding genes (*ACVR2A*, *ALB*, *CIDEB*, *FOXO1*, *GPAM* and *TNRC6B)* and one lncRNA *(NEAT1)* significantly enriched for acquired somatic variants. We hypothesised that given the selective advantage these variants must endow during disease progression, rare pathogenic variants of these regions, associated with features of liver disease, might be identified.

### 3.1 Specific somatic mutations in recurrently mutated genes are not found in the germline

We previously identified 129 unique variants across these six coding genes and one lncRNA (Table S2), the majority of which were either missense (49/129, 38%) or non‐coding exonic variants in *NEAT1* (49/129, 38%), and predicted to have a moderate or high impact upon protein structure (61/129, 47%, Table S3). Fifteen percent (11/71) of coding single‐nucleotide variants result in premature stops. 96% (51/53) of non‐synonymous coding variants had a CADD‐Phred^48^ score > 15, suggestive of deleterious impact on the protein (Table S4).

These variants are extremely rare in the germline, with 118/129 (92%) having never been identified across 150 463 individuals from gnomAD, 1000G, or the Exome Sequencing Project. The most common of these 11 previously reported variants was rs368997599 G > A (p.Arg45Trp) in *CIDEB*, which was identified in 10 individuals from gnomAD in the heterozygous state, seven of whom are of Ashkenazi Jewish ancestry. Even in this genetic ancestry p.Arg45Trp in *CIDEB* remained an ultra‐rare mutation with an allele frequency of 7.0 × 10^−4^. There was no evidence of associations between any of the previously identified variants and metabolic traits in the Common Metabolic Disease Portal (Table S5). None of the 11 previously identified variants were associated with significant eQTLs (Table S4).

Whilst all of the six coding genes had the expected number of missense mutations, four of the six (*ACVR2A*, *ALB*, *FOXO1* and *TNRC6B*) are under selection pressure to prevent against haploinsufficiency; in gnomAD there were significantly fewer predicted loss of function (pLoF) variants observed than expected (Table S3). This implies that there are a reduced number of germline variants in these genes that will cause pLoF, compared to other coding genes and potential haploinsufficiency in these genes is deleterious. The exception to this was *CIDEB*, where there was an expected number of missense and pLoF mutations, but no reports of these rare variants associated with liver phenotypes. Overall, the specific somatic mutations that we had previously identified are very rare in the germline, because of negative selection pressure and are not known to be associated with the development of liver disease.

### 3.2 Among recurrently mutated genes only germline exonic variation in *GPAM* is associated with liver phenotypes

Given the rarity (or absence) of these specific 129 variants in the germline, we investigated whether other rare variants at these loci were associated with liver disease (or related metabolic traits). This methodology combines the effect of multiple rare variants (e.g. loss of function or missense) within genes to account for the rarity of individual variants. We used data from https://genebass.org/ and https://azphewas.com/ (*n* = 281 852 from UK BioBank), which tests whether exonic germline variants either individually, or cumulatively using a gene‐based burden method, demonstrated an association with liver or metabolic phenotypes.

Analysis of individual coding variants found that p.Ile43Val in *GPAM* was associated with differences in serum lipids and the synonymous mutation 10–112 157 327‐T‐A (p.Pro681Pro) in *GPAM* was associated with ALT (Table 1). Exonic variants in other genes were associated with related metabolic traits, but not with liver disease phenotypes (Table 1 and Table S6). Using gene‐based burden testing, which adds together all variants within a single category (e.g. pLoF or missense), variants in *ALB* demonstrated associations with serum lipids, but no other markers of liver disease. Burden testing for pLoF variants in *TNRC6B* demonstrated a significant association with alcohol consumption habits, but no other markers of alcohol‐related disease (Table S6). Therefore, among the six recurrently mutated genes only rare germline coding variants in *GPAM* have been identified to be associated with serum lipid levels, rather than liver phenotypes.

**TABLE 1:** Top associations from rare variant and gene‐burden analyses

	Variant‐level analyses								
Gene	Phenotype	Variant	AA change	Allele frequency	Model	Beta	*p* value	Total	Source
*ACVR2A*	Creatinine	2‐147 899 548‐G‐A	p.Pro118Pro	0.30	genotypic	0.03	6.84E‐25	254 544	AZ
*GPAM*	Alanine aminotransferase	10–112 157 327‐T‐A	p.Pro681Pro	0.28	genotypic	0.03	9.11E‐19	255 248	AZ
*GPAM*	Alkaline phosphatase	10–112 157 327‐T‐A	p.Pro681Pro	0.28	dominant	0.04	2.61E‐24	255 341	AZ
*GPAM*	Apolipoprotein A	10–112 180 571‐T‐C	p.Ile43Val	0.27	genotypic	0.04	1.16E‐42	231 005	AZ
*GPAM*	Cholesterol	10–112 180 571‐T‐C	p.Ile43Val	0.27	genotypic	0.03	2.12E‐17	253 523	AZ
*GPAM*	Direct bilirubin	10–112 157 327‐T‐A	p.Pro681Pro	0.28	genotypic	0.02	1.20E‐08	217 133	AZ
*GPAM*	HDL cholesterol	10–112 180 571‐T‐C	p.Ile43Val	0.27	genotypic	0.04	3.95E‐43	232 376	AZ
*GPAM*	Hip circumference	10–112 180 571‐T‐C	p.Ile43Val	0.27	genotypic	−0.02	1.59E‐08	265 294	AZ
*GPAM*	LDL direct	10–112 180 571‐T‐C	p.Ile43Val	0.27	genotypic	0.02	1.03E‐09	253 059	AZ
*GPAM*	Leg fat mass (right)	10–112 180 571‐T‐C	p.Ile43Val	0.27	genotypic	−0.01	3.47E‐08	261 190	AZ
*GPAM*	Total bilirubin	10–112 157 327‐T‐A	p.Pro681Pro	0.28	genotypic	0.02	1.34E‐12	254 316	AZ
*GPAM*	Triglycerides	10–112 180 571‐T‐C	p.Ile43Val	0.27	genotypic	−0.02	2.07E‐13	253 317	AZ
*TNRC6B*	Body mass index (BMI)	22‐40 301 373‐A‐G	—	0.34	genotypic	−0.02	2.19E‐10	267 287	AZ
*TNRC6B*	Creatinine	22‐40 301 172‐TGCA‐T	p.Gln524del	0.30	genotypic	0.02	2.58E‐09	255 188	AZ
*TNRC6B*	Impedance of whole body	22‐40 301 373‐A‐G	—	0.34	genotypic	0.02	1.93E‐16	263 528	AZ
*TNRC6B*	Total bilirubin	22‐40 301 172‐TGCA‐T	p.Gln524del	0.30	genotypic	−0.02	3.87E‐08	254 308	AZ

	Gene burden analyses					
Gene	Phenotype	Burden test	Beta	*p*val	Total	Source
*ALB*	Apolipoprotein B	ptv	0.75	7.29E‐13	255 235	AZ
*ALB*	Cholesterol	pLoF	0.03	3.45E‐10	268 558	Genebass
*ALB*	Direct bilirubin	mis	0.003	1.86E‐08	228 253	Genebass
*ALB*	LDL direct	ptv	0.56	5.65E‐08	256 046	AZ
*ALB*	Total bilirubin	mis	0.003	1.60E‐11	267 473	Genebass
*TNRC6B*	Frequency of failure to fulfil normal expectations because of drinking alcohol in last year	pLoF	0.03	1.67E‐24	51 479	Genebass

*Note*: Summary statistics were obtained from analyses of individual rare variants (using UK BioBank 300 k Exomes) or gene‐burden testing for pLoF or missense variants. AA, amino acid; AZ, Astrazeneca PheWAS; mis, missense variants; pLoF, predicted loss of function; ptv, protein‐truncating variant; snv, single nucleotide variant. Significance threshold adjusted for multiplicity was *p* < 2.5 × 10^−8^ for GeneBass (using SKAT‐O test) and *p* < 2.0 × 10^−9^ (−log10[8.7]) for AZPheWAS.

### 3.3 Germline variation at *GPAM* , but not other somatically mutated genes, is associated with liver phenotypes

**FIGURE 1:**
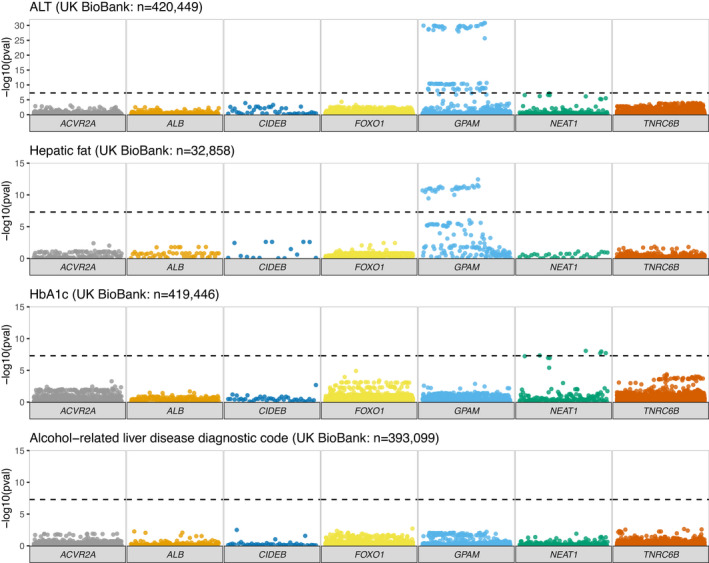
Association between common variants at recurrently mutated regions and markers of liver disease or glycaemic control. Manhattan plots focusing on the six protein‐coding genes and one lncRNA (*NEAT1*) of interest, illustrating all variants within their genomic coordinates. −log10 *p*‐value was obtained from summary statistics from the UK BioBank for alanine aminotransferase (ALT), liver fat (from Liu et al., 2021), glycosylated haemoglobin (HbA1c) and alcohol‐related liver disease (ARLD). Significance threshold adjusted for multiplicity was *p* < 5 × 10^−8^

**FIGURE 2:**
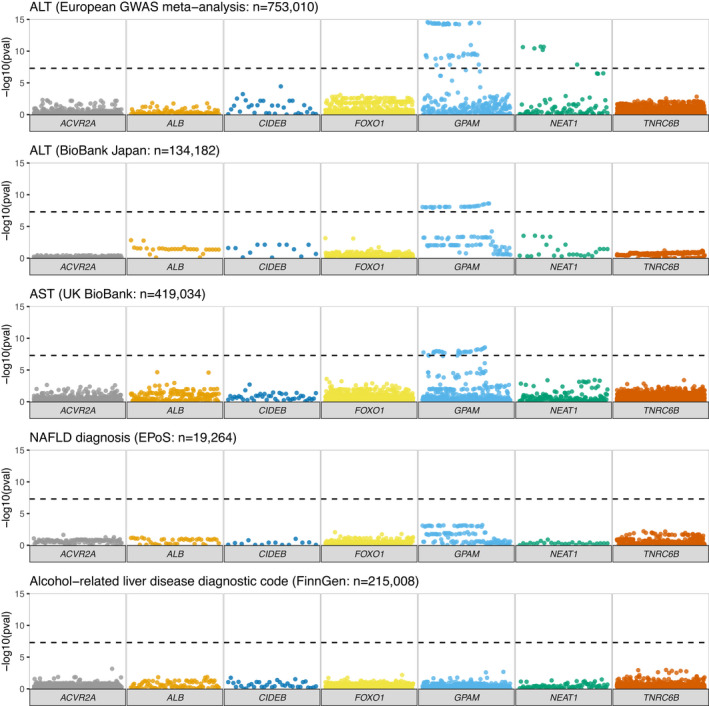
Association between common variants at recurrently mutated regions and markers of liver disease or glycaemic control. Manhattan plots focusing on the six protein‐coding genes and one lncRNA (*NEAT1*) of interest, illustrating all variants within their genomic coordinates. −log10 *p*‐value was obtained from summary statistics from the UK BioBank for aspartate aminotransferase (AST), hepatic fibrosis and cirrhosis. Data on diagnosis of NAFLD were obtained from Anstee et al. (2021). Significance threshold adjusted for multiplicity was *p* < 5 × 10^−8^

We next explored whether more common germline variants might be associated with liver phenotypes in previously published datasets. We utilised the summary statistics from the UK BioBank cohort of 420 000 subjects^33^ and searched for variants within these seven regions (Figures 1 and 2, Table 2 and Figure S1). As previously described,^19^, ^20^ we observed genome‐wide significant variants within *GPAM*, associated with elevated serum levels of ALT (Table 2, e.g. rs10787429 C > T beta = .006, *p* = 2.8 × 10^−39^ [*p*‐value significance threshold adjusted for multiplicity *p* < 5 × 10^−8^]), AST and liver fat by MR imaging (e.g. rs11446981 T > TA beta = −.003, *p* = 3.6 × 10^−13^). This acted as a useful positive control in our analyses that found no significant associations between SNPs near any of the other recurrently mutated coding genes and disease correlates in NAFLD or ARLD.

**TABLE 2:** Genome‐wide significant associations of common variants within regions of interest with eQTLs

Variant	Gene	Trait	Beta	*p* value	EAF	Source	*n*	eQTL gene (tissue)	eQTL beta (*p*‐value)	eQTL_study
rs10787429 C > T	*GPAM*	ALT	0.006	2.80E‐39	0.27	European GWAS meta‐analysis	753 010	Nil		
rs7096937 T > C	*GPAM*	AST	−0.02	2.77E‐09	0.73	UK BioBank	419 034	Nil		
rs11446981 T > TA	*GPAM*	Liver fat	−0.06	3.60E‐13	0.70	UK BioBank	32 858	Nil		
rs595366 T > A	*NEAT1*	ALT	−0.003	1.90E‐11	0.27	European GWAS meta‐analysis	753 010	NEAT1 (adipose)	−0.4 (*p* = 6.5 × 10^−17^)	FUSION
rs34743766 C > CA	*NEAT1*	HbA1c	0.01	8.49E‐09	0.17	UK BioBank	419 446	NEAT1 (adipose)	0.27 (*p* = 9.3 × 10^−17^)	TwinsUK

*Note*: Lead variants from the six protein‐coding genes and one lncRNA (*NEAT1*) of interest are associated with markers of liver disease or glycaemic control. GWAS summary statistics were obtained from the UK BioBank or Pazoki et al. (2021). Expression quantitative trait locus (eQTL) data were obtained from FIVEx. EAF, effect allele frequency. Significance threshold adjusted for multiplicity was *p* < 5 × 10^−8^.

We validated these associations across a European‐ancestry GWAS meta‐analysis (*n* = 753 010)^38^ and in the BioBank Japan cohort (*n* = 134182^37^; [*p*‐value significance threshold adjusted for multiplicity *p* < 5 × 10^−8^] Figure 2, Figure S1 and Table S7). The magnitude of effect size for the lead variants within *GPAM* was similar to that for well‐established loci in *HSD17B13* and *MTARC1*, but smaller than observed for risk variants in *PNPLA3* and *TM6SF2* (Table S8). For example, for change in hepatic fat^35^: 10–113 950 257‐T‐TA in *GPAM* beta = −.06, *p* = 3.60 × 10^−13^; compared to rs738409C > G in *PNPLA3*: beta = 0.22, *p* = 1.5 × 10^−133^ and rs58542926T > C in *TM6SF2* beta = 0.33, *p* = 2.5 × 10^−116^. Therefore, the only somatically mutated gene for which germline variation was associated with liver disease was *GPAM*.

Genome‐wide significant associations between variants in GPAM and fatty liver disease have been previously described, particularly for rs2792751T > C p.Ile43Val.^19^, ^20^ All the GPAM variants identified in the above analyses were non‐coding variants and in strong linkage disequilibrium (LD r^2^ > 0.93) with rs2792751T > C (Figure S2A).

Somatic variants in *GPAM* were associated with NAFLD and alcohol‐related liver disease (ARLD) in our previous study.^14^ Germline rs2792751T > C (p.Ile43Val) was also associated with ARLD or NAFLD diagnosis in these individuals (*p* = 0.002, Figure S3).

Unlike the somatic mutations in GPAM, p.Ile43Val is not predicted to have a major functional consequence (Table S4). rs2792751T > C (and non‐coding GPAM variants identified in this study) were only associated with significant reductions in GPAM mRNA in tibial artery tissue (Table S4). No significant eQTLs in liver were identified. We did not find evidence of significant associations between p.Ile43Val with clinical liver‐related events in the UKBB cohort, though the absolute number of cases was comparatively small (Table S9).

### 3.4 Germline variation at the long non‐coding RNA *NEAT1* is associated with liver phenotypes and glycaemic control

In the UK BioBank cohort, we observed genome‐wide significant variants within the lncRNA *NEAT1* for elevated serum ALT (Figure 1). This was replicated in the European‐ancestry ALT meta‐analysis, though not in the BioBank Japan ALT results (Figure 2). The lead variant for *NEAT1* (rs595366T > A) was also found to have an eQTL for *NEAT1* in both adipose and liver tissue. Germline variation at rs595366T > A was also associated with the diagnosis of ARLD or NAFLD in the small cohort of individuals characterised in Ng et al.^14^ (*p* = 0.002, Figure S3).

However, no associations were identified between variants at *NEAT1* and categorical definitions of liver disease (e.g. diagnosis of NAFLD or ARLD, Figures 1 and 2) or severity of liver disease (e.g. cirrhosis). This was consistent across data from the UK BioBank and FinnGen (Figure S1 and Tables S7 and S9) datasets.

Given the causal associations between insulin resistance and hepatic steatosis, we investigated whether variants were associated with HbA1c or a diagnosis of type 2 diabetes mellitus (T2DM). Variants in *NEAT1* were associated with HbA1c levels in the UK BioBank (e.g. rs34743766 C > CA, beta = 0.1, *p* = 8.5 × 10^−9^ [*p*‐value significance threshold adjusted for multiplicity *p* < 5 × 10^−8^]) and had an associated eQTL for *NEAT1* in adipose tissue, but this was not replicated for diagnosis of T2DM or other analyses of HbA1c (Figure S1). These associations are interesting as expression levels of *NEAT1* have previously been associated with both the presence of diabetes^49^ and the progression of diabetic complications.^49^, ^50^

All variants in or near NEAT1 identified to have significant genome‐wide association were in strong linkage disequilibrium with each other (LD *r* ^2^ > 0.82, Figure S2B). Many of the variants were found to have significant eQTLs for NEAT1 in a range of highly metabolically active tissues (Table 3), but not in the liver.

**TABLE 3:** Associations between common variants in or near regions of interest and related metabolic traits

Gene	Variant	Trait	Beta (SE)	*p* value	*n*	eQTL
*ACVR2A*	rs13008838 A > G	eGFR‐creat (serum creatinine)	−0.003 (0.0004)	4.97E‐15	754 661	ACVR2A: Adipose ‐ Subcutaneous (+), Muscle skeletal (+), Artery ‐ Tibial (+), Artery ‐ Aorta (+), Pancreas (+), Artery ‐ Coronary (+), Adipose ‐ Visceral (Omentum) (+)
*ACVR2A*	rs3764955 G > C	Serum creatinine	0.020 (0.003)	2.01E‐08	182 901	ACVR2A: Artery ‐ Coronary (+), Adipose ‐ Visceral (Omentum) (+), Adipose ‐ Subcutaneous (+), Pancreas (+), Artery ‐ Aorta (+), Artery ‐ Tibial (+), Muscle skeletal (+)
*CIDEB*	rs12590407 G > A	Diastolic blood pressure	−0.010 (0.002)	9.11E‐12	552 754	
*FOXO1*	rs2253001 A > T	Basal metabolic rate	0.010 (0.002)	1.03E‐08	331 307	
*GPAM*	rs10787429 T > C	Alanine transaminase	−0.030 (0.005)	3.44E‐09	141 341	GPAM: Artery ‐ Tibial (−)
*GPAM*	rs7898213 T > C	Alkaline phosphatase	−0.030 (0.004)	5.00E‐11	112 189	
*GPAM*	rs2792759 C > T	Bilirubin	−0.010 (0.001)	4.10E‐09	467 109	GPAM: Artery ‐ Tibial (−)
*GPAM*	rs2297991 T > C	HDL cholesterol	−0.030 (0.003)	5.21E‐20	191 159	GPAM: Artery ‐ Tibial (−)
*GPAM*	rs1129555 A > G	LDL cholesterol	0.030 (0.004)	1.00E‐15	‐	GPAM: Artery ‐ Tibial (−)
*GPAM*	rs1129555 A > G	Total cholesterol	0.030 (0.004)	2.00E‐18	‐	GPAM: Artery ‐ Tibial (−)
*GPAM*	rs2254537 T > A	Triglycerides	0.020 (0.002)	8.23E‐17	482 392	GPAM: Artery ‐ Tibial (−)
*NEAT1*	rs10896037 A > G	Chronic kidney disease	0.060 (0.009)	5.98E‐10	140 966	NEAT1: Adipose ‐ Visceral (Omentum) (−), Muscle skeletal (−), Adipose ‐ Subcutaneous (−), Artery ‐ Aorta (−)
*NEAT1*	rs12801636 G > A	Coronary artery disease	−0.040 (0.004)	6.74E‐17	1 546 260	
*NEAT1*	rs2306363 G > T	Diastolic blood pressure	−0.030 (0.002)	1.39E‐28	552 754	
*NEAT1*	rs4930319 G > C	eGFR‐creat (serum creatinine)	0.003 (0.0004)	8.69E‐27	757 454	NEAT1: Adipose ‐ Subcutaneous (−), Adipose ‐ Visceral (Omentum) (−), Artery ‐ Aorta (−), Muscle skeletal (−)
*NEAT1*	rs2236682 G > T	Serum creatinine	0.020 (0.004)	2.66E‐10	182 901	NEAT1: Muscle skeletal (−), Artery ‐ Aorta (−), Adipose ‐ Visceral (Omentum) (−), Adipose ‐ Subcutaneous (−)
*NEAT1*	rs2306363 G > T	Systolic blood pressure	−0.020 (0.002)	3.90E‐20	550 853	
*NEAT1*	rs10750766 C > A	Triglycerides	0.020 (0.002)	9.96E‐15	459 761	
*NEAT1*	rs947791 G > A	Type 2 diabetes	0.050 (0.004)	2.71E‐16	1 016 100	NEAT1: Artery ‐ Aorta (+), Pancreas (+), Muscle skeletal (+), Artery ‐ Tibial (+), Adipose ‐ Visceral (Omentum) (+), Adipose ‐ Subcutaneous (+)
*NEAT1*	rs11227217 C > T	Waist‐hip ratio	0.020 (0.002)	2.60E‐15	997 776	NEAT1: Adipose ‐ Visceral (Omentum) (+), Muscle skeletal (+), Adipose ‐ Subcutaneous (+), Artery ‐ Aorta (+), Artery ‐ Tibial (+), Pancreas (+)
*NEAT1*	rs4645917 G > A	Waist‐hip ratio adj BMI	−0.030 (0.002)	5.05E‐18	969 126	NEAT1: Muscle skeletal (−), Artery ‐ Tibial (−)
*TNCR6B*	rs4820410 A > G	BMI	−0.020 (0.001)	1.72E‐26	1 326 100	TNRC6B: Artery ‐ Tibial (+)
*TNCR6B*	rs2294352 G > A	eGFR‐creat (serum creatinine)	0.010 (0.001)	2.90E‐32	772 751	
*TNCR6B*	rs470113 A > G	Pulse pressure	0.020 (0.003)	4.33E‐08	317 539	TNRC6B: Artery ‐ Aorta (+), Artery ‐ Tibial (+), Artery ‐ Aorta (+), Artery ‐ Tibial (+)
*TNCR6B*	rs2294352 G > A	Serum creatinine	−0.030 (0.004)	1.06E‐15	191 380	
*TNRC6B*	rs5995840 T > C	Body mass index	0.030 (0.004)	1.02E‐11	173 430	TNRC6B: Artery ‐ Aorta (+), Artery ‐ Tibial (+), Atherosclerotic aortic root NA

*Note*: Gene‐based PheWAS performed using Phenoscanner and Common Metabolic Disease Portal to identify common variants significantly associated with the six protein‐coding genes and one lncRNA (*NEAT1*) of interest. eQTL column provides the gene that has significant eQTLs for that variant, the tissues and the direction (+, positive eQTL; −, negative eQTL). eQTL data were obtained using the Qtlizer package for R. Significance threshold adjusted for multiplicity was *p* < 5 × 10^−8^.

### 3.5 Germline variation at recurrently mutated genes is associated with metabolic phenotypes

We then explored whether common variants in or near these regions were associated with other related metabolic traits that affect patients with NAFLD, using a gene‐ (or region‐) based phenome‐WAS from two sources (Phenoscanner^29^ and Common Metabolic Disease Portal,^30^ *n* = 7637‐1 546 260, Table S10). In addition to its association with ALT, variants in or near *GPAM* influenced the concentration of many serum lipids (Table 3), including LDL cholesterol and triglyceride levels. We also identified variants near *NEAT1* that were associated with a diagnosis of T2DM, waist‐to‐hip ratio and had a significant eQTL for *NEAT1* in adipose tissue. In addition, variants in or near *TNRC6B* were associated with BMI and creatinine, as were those in *ACVR2A*. Therefore, germline variation in these genes may impact on related phenotypes that accompany the metabolic syndrome.

## 4 DISCUSSION

Investigating the genetics of fatty liver disease has the potential to inform our understanding of disease biology and facilitate clinical risk stratification for affected patients.^4^ We recently identified six protein‐coding genes and one lncRNA (*NEAT1*) that are enriched for loss of function somatic mutations in patients with NAFLD or ARLD.^14^ In this study, we find that germline variation in only one, *GPAM*, was robustly associated with markers of liver disease. The variants in *GPAM* are in strong linkage disequilibrium with a benign (or gain of function) variant: p.Ile43Val. This implies that although strong selection pressure exists to acquire loss of function mutations in existing fatty liver, we did not find evidence that similar germline variation contributes to disease initiation. This important negative result has important implications: (1) different pathophysiological mechanisms likely operate in fatty liver disease initiation (e.g. gain of function in *GPAM*) and progression driving stage‐specific selective advantage (e.g. loss of function); (2) genetic risk scores capturing only germline variation will not include the pathogenicity conveyed by these regions and additional strategies may be needed to understand the prognostic implications of these somatic mutations.

Our analysis has replicated the known associations between variants in or near *GPAM* with liver fat, as well as ALT and AST, acting as a positive ‘control’ for analyses of the other loci. These variants are likely proxies for the coding variant p.Ile43Val. This variant has no significant hepatic eQTLs and is not predicted to disturb the protein structure, therefore may cause a gain of function in GPAM. This is in marked contrast to the somatic mutants in *GPAM* identified by Ng et al.,^14^ all of which were predicted to be deleterious. Whilst we did not show these variants to influence the diagnosis of NAFLD, this has been demonstrated by others^21^ with larger sample sizes, variants in *GPAM* (particularly p.Ile43Val) are associated with radiological and histological diagnosis of NAFLD.^19^, ^20^, ^21^, ^22^ However, it is important to note that variants in *GPAM* have a comparatively small effect size compared to variants in *PNPLA3* and *TM6SF2*, but similar to those in *HSD17B13* and *MTARC1*.

For five (*ACVR2A*, *ALB*, *CIDEB*, *FOXO1*, *TNRC6B*) of the six protein‐coding genes under investigation in this study, we found no evidence for the association between germline variation and liver disease. These observations were consistent across multiple data sources, traits, genetic ancestries and analysis methodologies, for example both common variant analyses and rare‐variant gene burden testing. This was an unexpected observation, given the genetic evidence for their selective advantage in hepatocyte clones in diseased non‐malignant NAFLD and ARLD.^14^ Moreover, *CIDEB* and *FOXO1* have well‐established functions as a lipid droplet‐associated protein^15^ and a component of the insulin signalling cascade^17^ respectively. Conversely, our previous study did not identify acquired somatic mutations in genes with strong germline associations with liver disease (e.g. *PNPLA3*, *TM6SF2*). Collectively, this suggests that the influence of germline and acquired variants on parenchymal liver disease occurs through independent mechanisms (and genes). It should be noted that the methodology employed in this study does not exclude the possibility that individuals with rare loss‐of‐function mutations in these genes may have liver‐related phenotypes. Identification and studying human knock‐outs for these genes is an alternative strategy for investigating whether germline variation plays a role in liver disease, as has been illustrated for other conditions.^51^, ^52^ However, our data suggest that these individuals will be very rare.

We identified borderline associations between variants in *NEAT1*, a long non‐coding RNA (lncRNA), with ALT and HbA1c. Our broader analyses implicated *NEAT1* in influencing multiple metabolic traits (e.g. serum triglycerides, diagnosis of T2DM and coronary artery disease), that are of potential relevance to patients with NAFLD. These results point towards a primary role on insulin resistance, potentially through modulation of adipose tissue biology, as several variants in this lncRNA also had significant eQTLs in adipose tissue, but not in the liver. The biology of *NEAT1* is poorly understood, but there is some in vitro evidence for its role in adipogenesis.^53^ We suggest that the subtle effects of germline variants in *NEAT1* on ALT are likely indirect, via perturbation of insulin resistance and/or development of T2DM,^54^ however further work is required to establish this. These data also underline the principle that the genomic regions enriched for somatic mutations in our original analysis are principally those involved in metabolism.

We found that germline variation in lead variants in GPAM and NEAT1 was associated with the diagnosis of NAFLD or ARLD, using data from our previous study. Therefore, in this small cohort, we found enrichment of both somatic and germline variation in these two genomic regions.

Clinically, one aim of human genetics is to stratify patients into high‐ and low‐risk groups for disease progression using polygenic gene scores. Such an approach can identify individuals with a five‐fold increased risk of coronary artery disease.^55^ This would be of particular use for NAFLD and ARLD, both common conditions where only a minority of individuals progress to liver‐related clinical events. To date, there have been four PGS published for liver disease,^11^, ^12^, ^56^, ^57^ all derived using genome‐wide significant hits, and therefore none of our seven genomic regions of interest were included. If a genome‐wide PGS were derived,^58^ which included weighting from sub‐genome wide‐significant variants, then variants in or near *GPAM* would contribute. However, they would still receive comparatively minimal weighting compared to variants in *PNPLA3* and *TM6SF2*. More broadly, it is not clear how the magnitude of prognostic implication would compare for germline variation risk scores compared to somatic mutations, as the prognostic implication of these remains unknown. Our results illustrate that the integration of somatic mutants into prognostic tools will be a complex process and separate from existing methods for polygenic gene scores.

One limitation of this study is that rare variant associations may not be observed because of a lack of power. Larger population‐based datasets and disease‐specific cohort studies may in future identify links between variants and liver‐related outcomes that we have not been able to observe. In addition, we have not investigated evidence of interaction between genetic and environmental triggers (e.g. body mass index, alcohol consumption), as has been shown for other variants that influence liver fat.^59^

## 5 CONCLUSION

Out of seven genomic regions with selective pressure for acquired loss of function mutations secondary to NAFLD and ARLD, only germline variation in *GPAM* is predictive of liver disease. Unlikely somatic mutations, the lead coding variant in GPAM (p.Ile43Val) is not predicted to deleteriously affect protein structure. This suggests that different pathophysiological mechanisms occur in disease initiation and progression. Therefore, genes with pathogenic somatic mutations in NAFLD and ARLD are distinct from those that confer germline risk and would not be captured by polygenic risk scores. Novel approaches will be required to integrate somatic and germline variation with clinical variables for risk prediction algorithms. These observations may be refined when larger sample sizes facilitate observations of subtle in rare variants.

## FUNDING INFORMATION

JPM is supported by a Wellcome Trust fellowship (216329/Z/19/Z); MH is supported by a CRUK Advanced Clinician Scientist fellowship (C52489/A19924); CRUK‐OHSU Project Award (C52489/A29681) and CRUK Accelerator award to the HUNTER consortium (C18873/A26813).

## CONFLICT OF INTEREST

MH is a co‐inventor on a patent detailing the finding of recurrent somatic mutations in chronic liver disease.

## Supporting information


Figure S1

Figure S2

Figure S3

Table S1
Table S2
Table S3
Table S4
Table S5
Table S6
Table S7
Table S8
Table S9
Table S10
