# Supplementary material for: A minority of somatically mutated genes in pre‐existing fatty liver disease have prognostic importance in the development of NAFLD
Source: Liver Int. 2022 May 11;42(8):1823–35. doi: 10.1111/liv.15283 (PMC9544140; doi:10.1111/liv.15283)
Supplement: Supplementary file 4 — Table S1 Table S2 Table S3 Table S4 Table S5 Table S6 Table S7 Table S8 Table S9 Table S10 [file LIV-42-1823-s001.docx]

**Supp. Table 1:** Summary of data sources for genome-wide association study summary statistics used, including number of participants.

**Supp. Table 2:** List of all included somatic variants with full annotation from Variant Effect Predictor.

**Supp. Table 3**: Characteristics of variants identified as somatic mutants in non-malignant NAFLD and ARLD liver tissue. Features of 129 unique somatic mutants across six protein-coding genes and one lncRNA (*NEAT1*). Variants were annotated using Variant Effect Predictor to derive a predicted consequence and severity of impact. Germline frequency of variants was assessed across any of gnomAD, 1000G, and ESP (n=150,463). Data on predicted loss of function (pLoF), observed and expected (O/E) and missense variants was obtained from gnomAD v2.

**Supp. Table 4:** Annotation of coding single nucleotide variants with functional consequence predictions from dbNSFP.

**Supp. Table 5:** Associations between somatic variants previously identified in the germline and metabolic traits from the Common Metabolic Disease Portal. Critical p-value for significance adjusted for multiplicity was p<5.2x10^-4^.

**Supp. Table 6:** Full results from rare exonic variant and gene-burden analyses. Significance threshold adjusted for multiplicity was p<2.5x10^-8^ for GeneBass (using SKAT-O test) and p<2.0x10^-9^ (-log10(8.7)) for AZPheWAS.

**Supp. Table 7:** Full results from single common variant analysis for markers of liver disease from all studies (discovery and replication) across all regions of interest. Significance threshold adjusted for multiplicity was p < 5x10^-8^.

**Supp. Table 8:** Comparison of lead variants within GPAM region with well-established genome-wide significant variants across all studied traits. Significance threshold adjusted for multiplicity was p < 5x10^-8^.

**Supp. Table 10:** Associations between lead variants in *GPAM* and *NEAT1* with clinical liver-related events in the UKBB, using data from Phenoscanner. Significance threshold adjusted for multiplicity was p < 5x10^-8^.

**Supp. Table 10:** Full results from gene-based PheWAS for common variants for associated metabolic traits from Phenoscanner and Common Metabolic Disease Knowledge Portal. Significance threshold adjusted for multiplicity was p < 5x10^-8^.
